# Aerosol pH and Ion Activities of HSO_4_^–^ and SO_4_^2–^ in Supersaturated Single Droplets

**DOI:** 10.1021/acs.est.2c01378

**Published:** 2022-09-01

**Authors:** Meng Li, Hang Su, Guangjie Zheng, Uwe Kuhn, Najin Kim, Guo Li, Nan Ma, Ulrich Pöschl, Yafang Cheng

**Affiliations:** † Minerva Research Group, Max Planck Institute for Chemistry, 55128 Mainz, Germany; ‡ Multiphase Chemistry Department, Max Planck Institute for Chemistry, 55128 Mainz, Germany; § Institute for Environmental and Climate Research, Jinan University, Guangzhou 511443, China

**Keywords:** aerosol acidity measurement, ion activities, supersaturated single droplets, aerosol optical tweezers, Raman spectroscopy

## Abstract

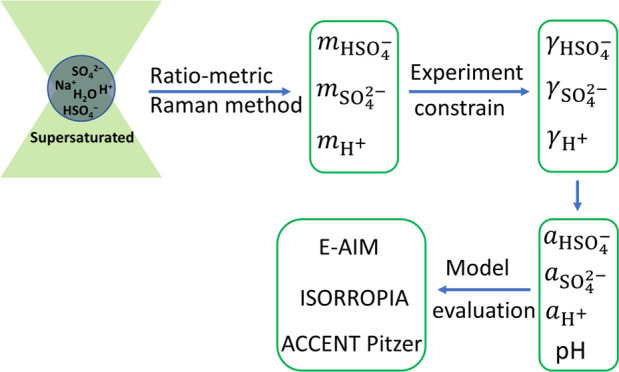

Accurate determination of acidity (pH) and ion activities in aqueous droplets is a major experimental and theoretical challenge for understanding and simulating atmospheric multiphase chemistry. Here, we develop a ratiometric Raman spectroscopy method to measure the equilibrium concentration of sulfate (SO_4_^2–^) and bisulfate (HSO_4_^–^) in single microdroplets levitated by aerosol optical tweezers. This approach enables determination of ion activities and pH in aqueous sodium bisulfate droplets under highly supersaturated conditions. The experimental results were compared against aerosol thermodynamic model calculations in terms of simulating aerosol ion concentrations, ion activity coefficients, and pH. We found that the Extended Aerosol Inorganics Model (E-AIM) can well reproduce the experimental results. The alternative model ISORROPIA, however, exhibits substantial deviations in SO_4_^2–^ and HSO_4_^–^ concentrations and up to a full unit of aerosol pH under acidic conditions, mainly due to discrepancies in simulating ion activity coefficients of SO_4_^2–^–HSO_4_^–^ equilibrium. Globally, this may cause an average deviation of ISORROPIA from E-AIM by 25 and 65% in predicting SO_4_^2–^ and HSO_4_^–^ concentrations, respectively. Our results show that it is important to determine aerosol pH and ion activities in the investigation of sulfate formation and related aqueous phase chemistry.

## Abstract 2

Accurate determination of aerosol pH and ion activities by ratiometric Raman analysis of levitated single droplets.

## Introduction

Aerosol acidity (pH) quantifies the activity of hydrogen ions (H^+^) in aqueous solution.^1^ It is a key parameter in atmospheric multiphase chemistry,^2^ influencing sulfate formation,^3−6^ secondary organic aerosol formation,^7−9^ phase partitioning,^10^ etc. Determining aerosol pH is thus essential for understanding and simulating the physicochemical processes of atmospheric aerosols, fine particulate matter, and their effects on climate and human health.^2,11−13^ There have been many attempts to estimate aerosol pH by thermodynamic equilibrium models.^11,14,15^ However, even using the state-of-the-art ISORROPIA^16^ and the Extended Aerosol Inorganics Model (E-AIM),^17^ the aerosol pH estimated from these two models can differ from each other by up to 1 pH unit.^11,15,18^ Given that the physicochemical processes of atmospheric aerosols are very sensitive to aerosol pH, e.g., 1 unit change in pH can completely change the dominant sulfate formation pathway^3,5,6,19^ and ice nucleation activity and mechanism,^20^ it is essential to accurately predict aerosol pH. However, it is currently not possible to pinpoint, which of the model results is closer to the truth due to the lack of sufficiently precise measurement data, especially in the supersaturated concentration range.^11^

Sulfate (SO_4_^2–^) is a major component of fine particulate matter in the atmosphere.^3,21^ Its equilibrium with bisulfate (HSO_4_^–^) was recently suggested to be an important underlying reason for the differences in aerosol pH predictions among different models.^11,15,22^ Moreover, this equilibrium should be the key to estimating the sulfate concentration in the atmosphere, especially in areas with high aerosol acidity. Therefore, direct experimental results are required to evaluate the performance of different models in treating SO_4_^2–^–HSO_4_^–^ equilibrium and further for pH predictions. Recently, several experimental approaches have been developed to determine aerosol pH, such as measurements using pH-indicator paper,^13,23−26^ UV–Vis spectrometry,^27−29^ and Raman microspectroscopy.^30−35^ Raman spectroscopy particularly has the capacity to measure ion concentrations of individual droplets.^30,31^ In these systems,^13,23−35^ aerosol droplets were collected on substrates before measurements. However, the deposition of droplets on substrates would hinder its application in atmospheric aerosols, since aerosol droplets are suspended in the atmosphere and can reach supersaturated conditions.^36,37^ At such high concentrations, phase transition/crystallization can occur on substrate contact, which makes measurements impossible or inaccurate.

Therefore, a contact-free method for measuring ion concentrations and pH of aerosol droplets is sorely needed to investigate a suspended aerosol system. Aerosol optical tweezers (AOT) have been used to trap particles through a strong gradient restoring force provided by a tightly focused laser beam.^38−40^ Coupled with Raman spectroscopy, aerosol fundamental properties such as composition, refractive index and size can be characterized.^38−40^ Recently, the AOT–Raman technique has been applied to determine the concentrations of conjugate acid–base to infer the pH of single trapped droplets.^41,42^ For example, Coddens et al.^42^ successfully used AOT–Raman to investigate the titration of aerosol pH via droplet coalescence. Boyer et al.^41^ combined the stimulated Raman peaks (whispering gallery modes, WGMs) and spontaneous Raman peaks to determine ion concentrations and further pH of sodium bisulfate (NaHSO_4_) droplets. However, in their study, both the determinations of ion concentrations and pH depended on E-AIM calculations. The droplet total solute (NaHSO_4_) concentrations were obtained from refractive index measurements (retrieved from WGMs) using the empirical correlation developed by Tang et al.^43^ The SO_4_^2–^ and HSO_4_^–^ concentration ratios were determined based on a calibration curve relating the spontaneous Raman peak area ratios to E-AIM calculated concentration ratios of these two ions. It was stated in Boyer et al.^41^ that the accurate droplet pH simulated by the E-AIM model was then constrained by both the total NaHSO_4_ concentration and the concentration ratio of SO_4_^2–^ and HSO_4_^–^. There, the role of the anion concentration ratio in the pH calculation is, however, unclear, as the mentioned web-based E-AIM model used in the study would only allow inputting the total solute concentration when calculating the pH of NaHSO_4_ droplets.

In this study, using AOT coupled with Raman spectroscopy, we developed a ratiometric Raman method that for the first time can accurately and directly measure the equilibrium concentrations of SO_4_^2–^ and HSO_4_^–^ ions in levitated individual droplets of NaHSO_4_ over a wide concentration range of 0.4–8.8 mol kg^–1^. Along with the charge balance constraint and experiment-based activity coefficients, the pH and ion activities of microdroplets can be quantified unambiguously. Moreover, we performed a comprehensive comparison between experimental results and model calculations to evaluate the performance of three thermodynamic models, i.e., the E-AIM model (version IV,^17^ referred to as E-AIM hereafter), the ACCENT Pitzer model^44−46^ (referred to as ACCENT hereafter), and the ISORROPIA model (version II^16^ hereafter abbreviated ISORROPIA), in terms of simulating pH, ion concentrations, and activity coefficients.

## Materials and Methods

### Materials

Sodium sulfate (Na_2_SO_4_, 99.0%) was purchased from Alfa Aesar and sodium bisulfate (NaHSO_4_, 95.0%) was purchased from Honeywell Fluka. Both chemicals were used as received without further purification. Deionized water (Millipore, Milli-Q, resistivity 18.2 MΩ cm) was used as the solvent to get different concentrations of Na_2_SO_4_ and NaHSO_4_ in aqueous solutions.

### AOT and Raman Spectrometers

The AOT–Raman system used in this work is a commercial one coupled with a cavity-enhanced Raman spectrometer (AOT-100, Brial). Aerosol microdroplets were generated by nebulizing standard solutions of NaHSO_4_ using a medical-grade nebulizer (OMRON MicroAIR U100) and were then introduced into the trapping cell. Droplets trapped by the focused trapping laser with a wavelength of 532 nm had radiuses of 5.5–9.2 μm in this study. The trapping cell is relative humidity (RH) controlled by mixing different ratios of dry and humidified N_2_ gas flows. The trapping power changed as a function of droplet size and was normally in the range of 25–200 mW. The trapping laser also acts as the Raman excitation light for chemical species within the droplet. Raman spectra were collected in the range of 330–1578 cm^–1^ for SO_4_^2–^ and HSO_4_^–^ and of 2972–3867 cm^–1^ for OH, with a 1 s acquisition time and a 1200 g mm^–1^ diffraction grating. The spectrometer was calibrated against a dual Hg–Ne/Ar USB lamp. Spontaneous Raman bands in the Raman spectra provide information about the droplet chemical composition while the stimulated Raman peaks (WGMs) provide information about the droplet size and refractive index with high precision.^47,48^

### Ratiometric Calibrations for SO_4_^2–^ and HSO_4_^–^

**Figure 1:**
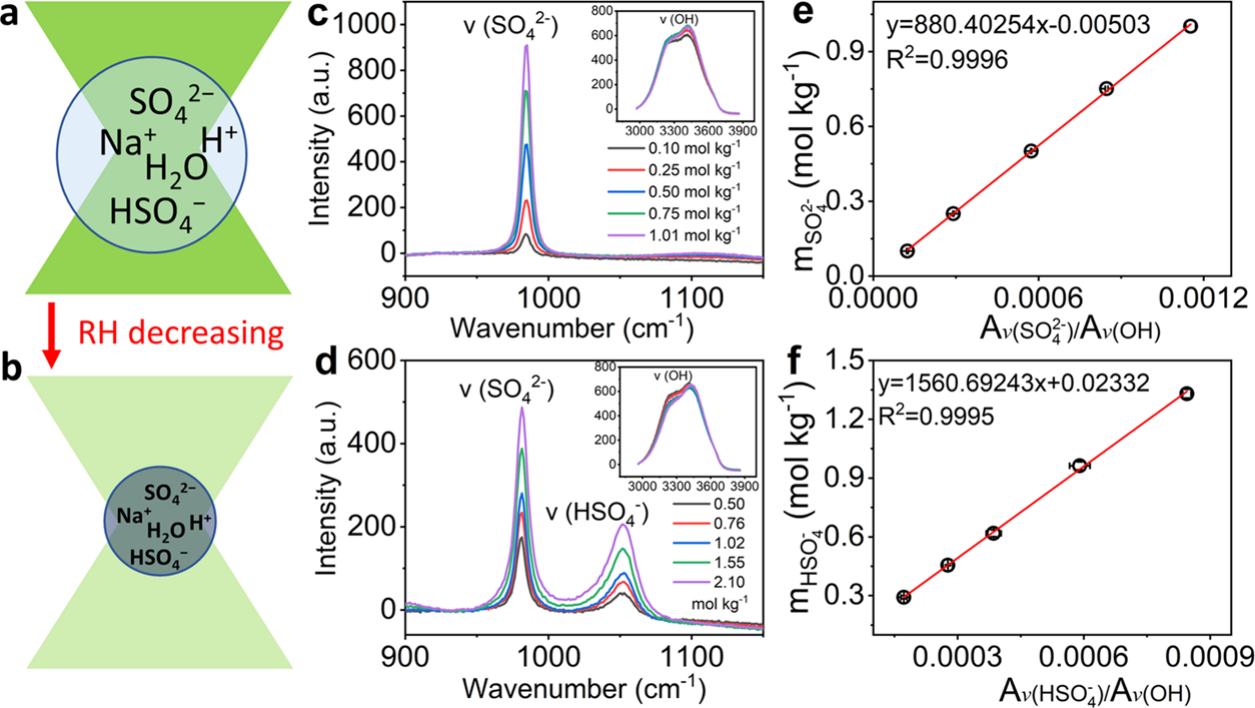
Ratiometric Raman analysis. (a, b) Schematic illustration of levitated single NaHSO_4_ droplets in aerosol optical tweezers with a decrease in relative humidity (RH). The droplet size decreases with the decrease in RH. Correspondingly, the solute concentration increases (dark blue) while the laser intensity decreases (lighter green). Raman detection volume of the particle also decreases relatively. Raman spectra of standard solutions of (c) Na_2_SO_4_ and (d) NaHSO_4_ with insets showing the OH band range. Calibration curves relating (e) SO_4_^2–^ molality (*m*_SO_4_^2–^_) to the integrated peak area ratio of SO_4_^2–^ and OH (*A*_v(SO_4_^2–^)_/*A*_v(OH)_) and (f) HSO_4_^–^ molality (*m*_HSO_4_^–^_) to the integrated peak area ratio of HSO_4_^–^ and OH (*A*_v(HSO_4_^–^)_/*A*_v(OH)_). The data points and error bars are the arithmetic mean values and standard deviations of three replicate measurements.

In the AOT–Raman system, the trapping laser of AOT acts as the Raman excitation light for chemical species within the droplet. As illustrated in Figure 1, since trapping power changes as a function of droplet size,^47,49^ not only the Raman detection volume but also the Raman excitation laser intensity differs considerably for droplets with different sizes. These variations make it impossible to directly determine ion concentrations from their respective Raman peak areas, as they are also droplet size dependent. In this study, Raman spectra were collected both in the fingerprint range for SO_4_^2–^ and HSO_4_^–^, and the OH signal range (Figure 1c,d). Instead of the absolute peak areas of SO_4_^2–^ (*A*_v(SO_4_^2–^)_) and HSO_4_^–^ (*A*_v(HSO_4_^–^)_), we applied the Raman peak area ratios of SO_4_^2–^ and OH (*A*_v(SO_4_^2–^)_/*A*_v(OH)_), and HSO_4_^–^ and OH (*A*_v(HSO_4_^–^)_/*A*_v(OH)_) for the calibration and determination of SO_4_^2–^ and HSO_4_^–^ concentrations, respectively (Figure 1e,f). This ratiometric approach offers two key advantages. First, due to the normalization by the OH signal of water, the apparent area ratios are insensitive to the influence of varying droplet sizes, detection volumes, and laser intensity in the AOT–Raman system. Second, the peak area ratios are directly related to SO_4_^2–^ concentrations (*m*_SO_4_^2–^_) and HSO_4_^–^ concentrations (*m*_HSO_4_^–^_) in units of molality (mol kg^–1^), i.e., the molar amount of solute per mass unit of solvent, which can be directly used to calculate H^+^ concentrations (*m*_H^+^_) (further for pH calculations) and evaluate model performance. Note, in the study reported by Coddens et al.,^42^ they used peak areas to make the calibration curves for ion concentrations, as they chose droplets with the same diameter, which can avoid the issues of detection volumes and laser intensities of droplets. However, this approach cannot solve the detection volume issue between droplets and bulk solutions, as the detection volume of bulk solutions is significantly larger than that of droplets, suggesting that bulk solutions with known ion concentrations cannot be used to create calibration curves for ions within droplets. The authors thus used droplets to make calibration curves by assuming that the ion concentration in the trapped droplet is the same as in the bulk solution. However, this assumption may not be always true, as the concentration in droplets is controlled by the RH inside the trapping optical cell.^36^

Standard solutions of Na_2_SO_4_ and NaHSO_4_ were used to generate calibration curves relating *m*_SO_4_^2–^_ and *m*_HSO_4_^–^_ to integrated Raman peak area ratios of SO_4_^2–^ and OH (*A*_v(SO_4_^2–^)_/*A*_v(OH)_) and HSO_4_^–^ and OH (*A*_v(HSO_4_^–^)_/*A*_v(OH)_), respectively (Figure 1e,f). The calibration measurements were performed by adding 100 μL of solution onto a coverslip (0.12 mm thickness, Paul Marienfeld GmbH & Co. KG) placed over the objective that focuses the 532 nm excitation laser of the AOT–Raman system with the laser power set to 50 mW. The peaks at 981 and 1050 cm^–1^ originate from the stretching vibrations of SO_4_^2–^ and HSO_4_^–^, respectively,^50−52^ and the broad bands centered at around 3400 cm^–1^ are related to the OH stretching of water^53,54^ (Figure 1c,d). The upper and lower limits of integration for the OH band were set to 3000 and 3850 cm^–1^ with the LARA program of AOT. For SO_4_^2–^ and HSO_4_^–^ peaks, Origin 2018 software was used to fit and integrate the peak areas in the range of 915–1105 cm^–1^. The SO_4_^2–^ calibration curve generated with Na_2_SO_4_ was used to determine *m*_SO_4_^2–^_ in the NaHSO_4_ solutions and further used to generate the HSO_4_^–^ calibration curve considering the stoichiometric relation *m*_NaHSO_4__ = *m*_SO_4_^2–^_ + *m*_HSO_4_^–^_. The molarities of the standard solutions were converted into molality units using the solution density determined by a model in the literature.^55^

### Droplet pH Determination

The pH value is defined as the H^+^ activity (*a*_H^+^_) in an aqueous solution^14^
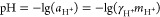
M1where γ_H^+^_ is the molality-based H^+^ activity coefficient and its determination is discussed in the Results and Discussion section. *m*_H^+^_ is the molality of dissociated H^+^. Fifty-eight micrometer-sized single NaHSO_4_ droplets were measured using the AOT–Raman system. For each investigated droplet, these quantities were determined as follows. From the measured Raman spectra, *m*_SO_4_^2–^_ and *m*_HSO_4_^–^_ were determined using the ratiometric calibration curves, and *m*_H^+^_ is determined based on the charge balance of NaHSO_4_ droplets
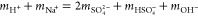
M2where the concentration of Na^+^ (*m*_Na^+^_) equals the sum of *m*_SO_4_^2–^_ and *m*_HSO_4_^–^_ (total concentration of sulfur) according to the stoichiometric formula of NaHSO_4_. In addition, as NaHSO_4_ droplets are strongly acidic, the concentration of OH^–^ (*m*_OH^–^_) can be neglected. Therefore, *m*_H^+^_ can be determined by
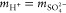
M3

### Thermodynamic Model Calculations

The experimental results were compared against three aerosol thermodynamic models that are often used to estimate ion equilibrium concentrations, activity coefficients, and pH of atmospheric aerosols: E-AIM,^17^ ACCENT,^44−46^ and ISORROPIA.^16^ The inputs for E-AIM (http://www.aim.env.uea.ac.uk/aim/model4/model4c.php) and ACCENT (http://www.aim.env.uea.ac.uk/aim/accent4/model.php) were the temperature (298.15 K) and the total molality of NaHSO_4_ (*m*_NaHSO_4__ = *m*_SO_4_^2–^_ + *m*_HSO_4_^–^_) as determined by the AOT–Raman methods for each investigated droplet. The outputs were the equilibrium concentrations, activity coefficients for each ion, and the equilibrium RH. Note, direct output activity coefficients of E-AIM are mole fraction-based activity coefficients (*f*), which were further converted to molality-based ones (γ) by γ = *fx*_w_ (*x*_w_ is the mole fraction of water, one of the E-AIM outputs).^56^ The molality-based ion activity coefficients are used throughout this work. For ISORROPIA, the forward and metastable mode was used, with inputs of temperature, total NaHSO_4_ concentration, and RH (as obtained from E-AIM), and outputs of equilibrium ion concentrations and mean activity coefficients. For each model, the aerosol pH was calculated from γ_H^+^_ and *m*_H^+^_ using eq M1.

When comparing ion activity coefficients, for the measurements, the ion activity coefficients were determined from the HSO_4_^–^ dissociation equilibrium of
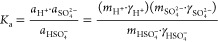
M4where *K*_a_ is the HSO_4_^–^ dissociation constant, which is 0.01 at 298 K^30^ and γ*_X_* is the ion activity coefficient of *X*. Combined with the charge balance of the NaHSO_4_ system (eq M3), the ion activity coefficients involved in the HSO_4_^–^ dissociation equilibrium can be directly calculated by
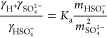
M5where *m*_SO_4_^2–^_ and *m*_HSO_4_^–^_ are experimentally measured values. For the three thermodynamic models, E-AIM and ACCENT calculate single-ion coefficients using PSC^56,57^ and the Pitzer activity coefficient model,^45^ respectively. Therefore, the expression of ion activity coefficients involved in the HSO_4_^–^ dissociation equilibrium (the left part of eq M5) can be determined from the model calculated γ_H^+^_, γ_SO_4_^2–^_ and γ_HSO_4_^–^_. While ISORROPIA calculates binary mean activity coefficients for the cation–anion pairs based on the Kusik and Meissner model in combination with the Bromley rule,^58^ and the single-ion activity coefficient product in the dissociation equilibrium of HSO_4_^–^ was expressed by mean activity coefficients in the form of^59^
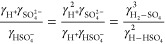
M6

### Calculation of Global Aerosol pH and Ion Concentrations

The global ion concentrations of Na^+^, SO_4_^2–^, NH_4_^–^, NO_3_^–^, Cl^–^, Ca^2+^, K^+^ and Mg^2+^ are calculated using the global GEOS-Chem model at a resolution of 2.5° longitude × 2° latitude with 47 vertical layers for 2016. Detailed model settings are provided elsewhere.^12^ These annual average ion concentrations, RH, and temperature were used to estimate aerosol pH and equilibrium by E-AIM and ISORROPIA. Both models were run in the forward and metastable mode. Since E-AIM cannot treat the crustal species, the presence of those species was accounted for using the charge-equivalent amount of Na^+^, i.e., 1 mol of K^+^ was replaced by 1 mol of Na^+^ and 1 mol of Mg^2+^ or Ca^2+^ was replaced by 2 mol of Na^+^. To avoid the influence of different crustal ions, we have made the same treatment for both E-AIM and ISORROPIA. So, the comparison between E-AIM and ISORROPIA in our study is not influenced by treating crustal species with Na^+^. In addition, we evaluated the uncertainties induced by treating the crustal species using the charge-equivalent amount of Na^+^ using ISORROPIA. We calculated the pH and ion concentrations of global PM_2.5_ in the presence of crustal species and with crustal species replaced by the charge-equivalent amount of Na^+^, respectively. We found that the deviations caused by treating the crustal species using the charge-equivalent amount of Na^+^ are on average 0.17 pH unit for pH, 0.05 μg m^–3^ for SO_4_^2–^ concentrations, and 0.004 μg m^–3^ for HSO_4_^–^ concentrations.

## Results and Discussion

### Ion Concentrations

**Figure 2:**
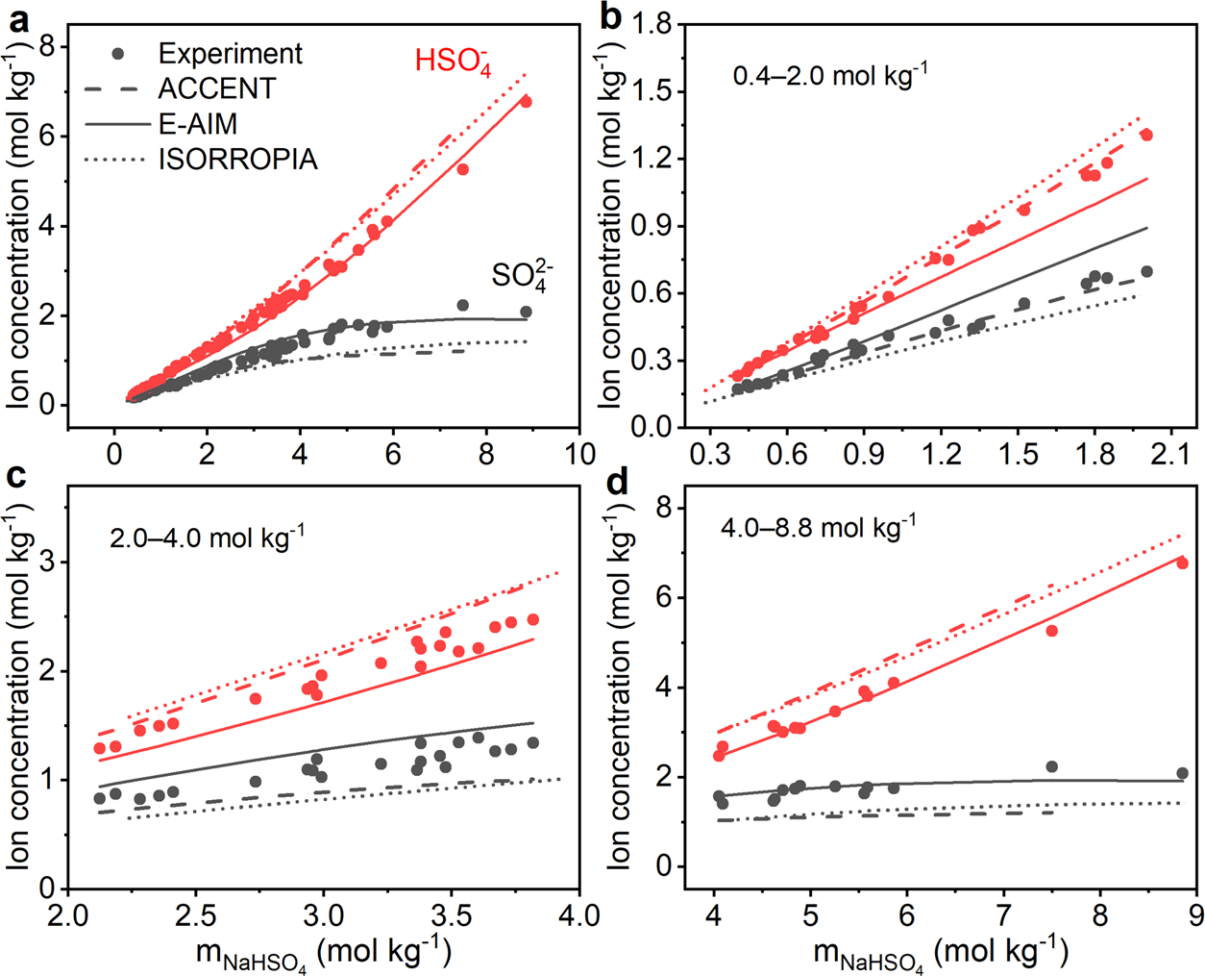
Experimentally measured and model calculated ion concentrations of single droplets. (a) *m*_SO_4_^2–^_ (black) and *m*_HSO_4_^–^_ (red) determined from direct droplet measurements (solid circle), ACCENT (dashed line), E-AIM (solid line), and ISORROPIA (dotted line) as a function of *m*_NaHSO_4__ of measurements or each model output. The expanded view of the ion concentrations with *m*_NaHSO_4__ ranging from (b) 0.4 to 2.0 mol kg^–1^, (c) 2.0 to 4.0 mol kg^–1^, and (d) 4.0 to 8.8 mol kg^–1^.

The *m*_SO_4_^2–^_ and *m*_HSO_4_^–^_ were measured in AOT–Raman experiments with over 50 micrometer-sized single droplets of aqueous NaHSO_4_ covering a concentration range of 0.4–8.8 mol kg^–1^ (Figure 2a, filled circles). The total NaHSO_4_ concentrations (*m*_NaHSO_4__) determined by our ratiometric Raman analysis agreed well with those calculated from droplet refractive indexes using the method developed by Tang et al.^43^ (Figure S1). The good agreement between the two independent methods validates the feasibility of extrapolating the calibration curves created from dilute bulk solutions to determine ion concentrations in droplets with high solute concentrations. It is worth noting that the NaHSO_4_ concentration range of 2.4–8.8 mol kg^–1^ in our system is unique in supersaturated droplets at 25 °C, as the concentration of bulk solution is limited by the solubility of NaHSO_4_ in water (≤2.4 mol kg^–1^ at 25 °C^60^). Therefore, our results can be used to evaluate thermodynamic model performances in predicting equilibrium ion concentrations for an aqueous HSO_4_^–^ system, especially at a supersaturated concentration range that cannot be done with bulk measurements.

Comparing the measured *m*_SO_4_^2–^_ and *m*_HSO_4_^–^_ with model simulations, we find that the three aerosol thermodynamic models (E-AIM, ACCENT, and ISORROPIA) showed different superiority in different *m*_NaHSO_4__ ranges (Figures 2 and S2). E-AIM overall agreed with measured ion concentrations, with an average relative deviation of 12.4% for *m*_SO_4_^2–^_ and 6.9% for *m*_HSO_4_^–^_ over the whole investigated *m*_NaHSO_4__ range (0.4–8.8 mol kg^–1^). A closer inspection shows that E-AIM calculated higher *m*_SO_4_^2–^_ and lower *m*_HSO_4_^–^_ in the relatively low *m*_NaHSO_4__ range (1.0–4.0 mol kg^–1^) compared with observations, with relative deviations ranging from 5.0 to 31.7% (average of 18.5%) for *m*_SO_4_^2–^_ and 3.3 to 15.9% (average of 10.2%) for *m*_HSO_4_^–^_. While it showed excellent agreement with the measurements under high *m*_NaHSO_4__ conditions (4.0–8.8 mol kg^–1^) as well as very low *m*_NaHSO_4__conditions (0.4–1.0 mol kg^–1^), with low relative deviations of *m*_SO_4_^2–^_ (average of 6.0%, 0.1–15.5%) and *m*_HSO_4_^–^_ (average of 3.3%, 0.1–7.8%).

Different from E-AIM, ACCENT showed substantial deviations under relatively high *m*_NaHSO_4__ conditions (2.0–8.8 mol kg^–1^), as indicated by the considerably underestimated *m*_SO_4_^2–^_ (relative deviations ranging from 3.7 to 45.7%, average of 23.9%) and overestimated *m*_HSO_4_^–^_ (relative deviations ranging from 2.0 to 22.7%, average of 13.0%). However, it showed excellent agreement with measurements in the low *m*_NaHSO_4__ range (0.4–2.0 mol kg^–1^), with low relative deviations of *m*_SO_4_^2–^_ (<14%, average of 6.0%) and *m*_HSO_4_^–^_ (<11%, average of 4.1%). ISORROPIA behaved similar to ACCENT. Compared with ACCENT, ISORROPIA was slightly closer to the measurements when *m*_NaHSO_4__ is larger than 4.0 mol kg^–1^, while deviated more from the measurements when *m*_NaHSO_4__ is less than 4.0 mol kg^–1^. Note, in the low *m*_NaHSO_4__ range (0.4–2.0 mol kg^–1^), although ISORROPIA predictions looked quite close to the corresponding experimental results in Figure 2, the relative deviations reach up to 40% for *m*_SO_4_^2–^_ and 44% for *m*_HSO_4_^–^_ (Figure S2). This apparent inconsistency is due to ISORROPIA’s insensitivity to small RH changes, which is discussed in detail in the Supporting Information (Text S1 and Figures S3 and S4).

### Ion Activities and Activity Coefficients

**Figure 3:**
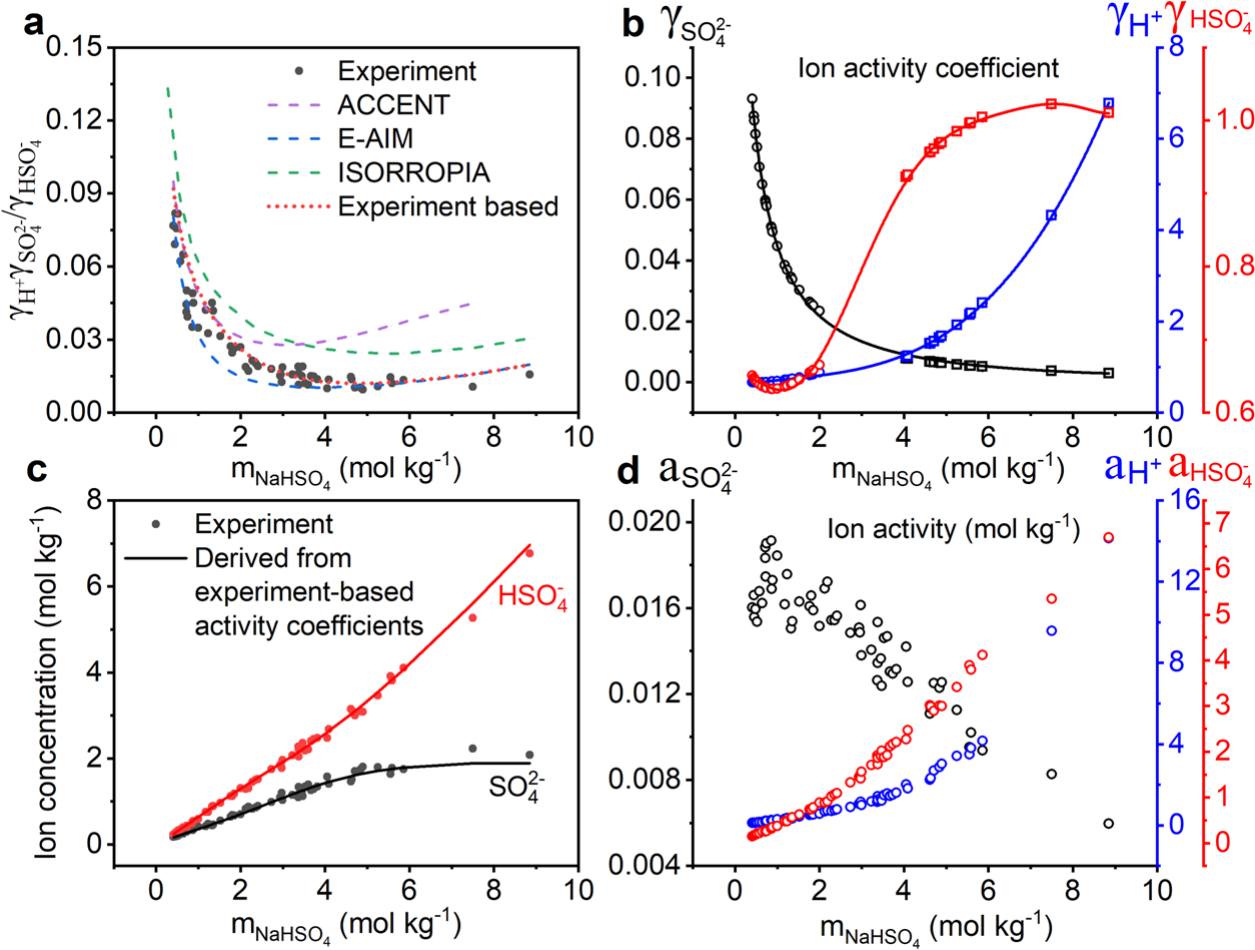
Observed and modeled ion activity coefficients, ion concentrations, and activities. (a) γ_H^+^_γ_SO_4_^2–^_/γ_HSO_4_^–^_ determined from measurements (black filled circle), ACCENT (purple dashed line), E-AIM (blue dashed line), ISORROPIA (green dashed line), and experiment-based ion activity coefficients (γ_H^+^_, γ_SO_4_^2–^_ and *m*_HSO_4_^–^_, which were determined from their corresponding fitting in b) (red dotted line) as a function of *m*_NaHSO_4__ of each method output. γ*_X_* is the ion activity coefficient of *X*. (b) Experiment-based γ_H^+^_ (blue line), γ_SO_4_^2–^_ (black line), and γ_HSO_4_^–^_ (red line). These experiment-based ion activity coefficients were obtained by fitting the ion activity coefficients calculated using ACCENT (circle) in the low *m*_NaHSO_4__ range (0.4–2.0 mol kg^–1^) and E-AIM (square) in the high *m*_NaHSO_4__ range (4.0–8.8 mol kg^–1^). The fitting equation is *y* = 0.58778 + 0.15503*x* – 0.04512*x*^2^ + 0.01206*x*^3^ (*R*^2^ = 0.9996) for γ_H^+^_, *y* = 1/(4.05908 + 15.64821*x* + 2.64213*x*^2^) (*R*^2^ = 0.9993) for γ_SO_4_^2–^_ and *y* = 0.64266 + 0.05639*x* – 0.16686*x*^2^ + 0.1352*x*^3^ – 0.04165*x*^4^ + 0.00623*x*^5^ – 4.58267 × 10^–4^*x*^6^ + 1.32966 × 10^–5^*x*^7^ (*R*^2^ = 0.9996) for γ_HSO_4_^–^_. (c) *m*_SO_4_^2–^_ (black) and *m*_HSO_4_^–^_ (red) determined from direct droplet measurements (filled circle) and from experiment-based γ_H^+^_, γ_SO_4_^2–^_ and γ_HSO_4_^–^_ (solid line). (d) Activities of H^+^ (blue), SO_4_^2–^ (black), and HSO_4_^–^ (red) determined from measured ion concentrations and experiment-based ion activity coefficients. Experimental *m*_H^+^_ was calculated by stoichiometric charge balance equation of aqueous NaHSO_4_ (eq M3).

Good performance of thermodynamic models in calculating ion concentrations often relies on accurate predictions of ion activity coefficients.^61^ Thus, in Figure 3a we compare the ion activity coefficients (γ_H^+^_γ_SO_4_^2–^_/γ_HSO_4_^–^_) calculated by thermodynamic models (dashed lines) with measured values (filled circles) (eqs M4–M6) at different NaHSO_4_ concentrations. It shows that ISORROPIA yielded markedly higher γ_H^+^_γ_SO_4_^2–^_/γ_HSO_4_^–^_ values over the whole *m*_NaHSO_4__ range (0.4–8.8 mol kg^–1^). As implied by eq M5, the overestimation of γ_H^+^_γ_SO_4_^2–^_/γ_HSO_4_^–^_ values would result in underestimated *m*_SO_4_^2–^_ and overestimated *m*_HSO_4_^–^_. This result is in good agreement with the comparison of measured and predicted ion concentration measurements (Figure 2), where ISORROPIA estimated substantially lower *m*_SO_4_^2–^_ and higher *m*_HSO_4_^–^_ compared with measurements. In the low *m*_NaHSO_4__ range (0.4–2.0 mol kg^–1^), ACCENT agreed well with the experimental results, consisting of its good predictions of ion concentrations. At high *m*_NaHSO_4__ conditions (4.0–8.8 mol kg^–1^), E-AIM was the closest to the measurements, in line with its excellent performance in ion concentration calculations. In the middle *m*_NaHSO_4__ range (2.0–4.0 mol kg^–1^), the measurement lies in between the prediction of ACCENT and E-AIM, corresponding to the underestimated *m*_SO_4_^2–^_ (overestimated *m*_HSO_4_^–^_) using ACCENT and overestimated *m*_SO_4_^2–^_ (underestimated *m*_HSO_4_^–^_) by E-AIM.

Considering the excellent performance of ACCENT in the low *m*_NaHSO_4__ range (0.4–2.0 mol kg^–1^) and E-AIM in the high *m*_NaHSO_4__ range (4.0–8.8 mol kg^–1^) (Figure 3a), the three single-ion activity coefficients (i.e., γ_H^+^_, γ_SO_4_^2–^_ and *m*_HSO_4_^–^_) calculated by these two models in the corresponding *m*_NaHSO_4__ range were fitted to obtain the experiment-based best estimation of γ_H^+^_, γ_SO_4_^2–^_ and γ_HSO_4_^–^_ over the whole *m*_NaHSO_4__ range of 0.4–8.8 mol kg^–1^ (Figure 3b). These experiment-based ion activity coefficients were used to recalculate the equilibrium ion concentrations in NaHSO_4_ droplets (see details in Text S2). As shown in Figure 3c, the predicted *m*_SO_4_^2–^_ and *m*_HSO_4_^–^_ (solid line) show excellent agreement with measurements, with a low average relative deviation of 6.2% for *m*_SO_4_^2–^_ and 3.8% for *m*_HSO_4_^–^_ over a wide *m*_NaHSO_4__ range of 0.4–8.8 mol kg^–1^ (Figure S5).

With the measured ion concentrations and experiment-based ion activity coefficients, the activities of H^+^ (*a*_H^+^_), SO_4_^2–^ (*a*_SO_4_^2–^_), and HSO_4_^–^ (*a*_HSO_4_^–^_) were determined (Figure 3d). *a*_H^+^_ and *a*_HSO_4_^–^_ increase substantially with *m*_NaHSO_4__, which is arising from the increase in both the corresponding ion concentrations and ion activity coefficients. Contrary to *a*_H^+^_ and *a*_HSO_4_^–^_, *a*_SO_4_^2–^_ decreases significantly with *m*_NaHSO_4__, due to the dramatic decrease of γ_SO_4_^2–^_, despite the slight increase of *m*_SO_4_^2–^_. Although *m*_H^+^_ and *m*_SO_4_^2–^_ always have the same value in NaHSO_4_ droplets, *a*_H^+^_ can be 3 orders of magnitude higher than *a*_SO_4_^2–^_ when *m*_NaHSO_4__ reaches ∼7.5 mol kg^–1^. These results demonstrate the significance of applying ion activities instead of ion concentrations when treating the equilibrium dissociation of HSO_4_^–^ in aerosol systems typically with high ionic strength.

### Droplet pH

**Figure 4:**
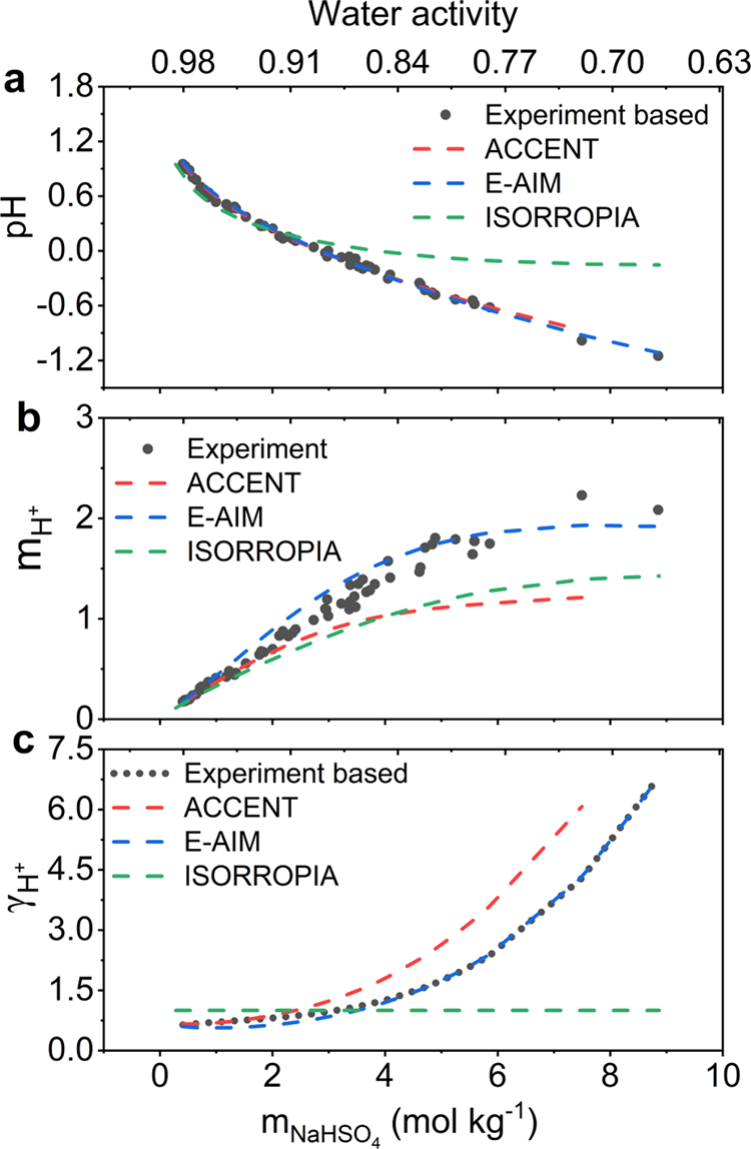
Comparison of observed and modeled *m*_H^+^_, γ_H^+^_, and pH. (a) Droplet pH, (b) *m*_H^+^_ and (c) γ_H^+^_ determined from experiments (black solid circle or dotted line), ACCENT (red dashed line), E-AIM (blue dashed line), and ISORROPIA (green dashed line) as a function of *m*_NaHSO_4__ of measurements or each model outputs and water activity calculated by E-AIM. Experimental *m*_H^+^_ was calculated by stoichiometric charge balance equation of aqueous NaHSO_4_ (eq M3).

The pH values of individual NaHSO_4_ droplets determined in AOT–Raman experiments cover the range from −1.15 to 0.95, with *m*_H^+^_ and γ_H^+^_ ranging from 0.17 to 2.23 mol kg^–1^ and 0.64 to 6.79, respectively (Figure 4). Droplet pH values were also calculated from thermodynamic models and the modeled results were compared with the measurements (Figures 4 and S6). E-AIM yielded very similar results, with small differences in estimated pH, *m*_H^+^_, and γ_H^+^_ in the wide *m*_NaHSO_4__ range of 0.4–8.8 mol kg^–1^. Specifically, the differences (E-AIM–experiment) in pH, log_10_*m*_H^+^_, and log_10_γ_H^+^_ are in the range from −0.07 to 0.09, −0.06 to 0.12, −0.11 to 0.006 pH units, respectively. Good agreements were also observed between ACCENT estimated pH values and the experiment-based ones, with a pH difference ranging from −0.07 to 0.11 pH units. Regarding *m*_H^+^_ and γ_H^+^_, small differences were observed in the low *m*_NaHSO_4__ range (0.4–2.0 mol kg^–1^), with the Δlog_10_*m*_H^+^_ ranging from −0.06 to 0.03 and a Δlog_10_γ_H^+^_ from −0.01 to 0.03. There are greater differences in *m*_H^+^_ and γ_H^+^_ predictions under high *m*_NaHSO_4__ conditions (2.0–8.8 mol kg^–1^). Compared to measured values, lower *m*_H^+^_ (−0.26 ≤ Δlog_10_*m*_H^+^_ ≤ −0.05) and higher γ_H^+^_ (0.04 ≤ Δlog_10_γ_H^+^_ ≤ 0.18) were estimated using ACCENT, leading to similar pH values to the observed ones. The Aerosol Inorganic–Organic Mixtures Functional groups Activity Coefficient model (AIOMFAC, https://aiomfac.lab.mcgill.ca)^62,63^ is another thermodynamic model that can calculate ion concentrations and ion activity coefficients. Overall it yielded similar results as E-AIM, with differences from the experimental results (AIOMFAC–experiment) in the range of 0.03–0.15 pH units for pH, −0.08 to 0.12 for log_10_*m*_H^+^_, and −0.19 to 0.03 for log_10_γ_H^+^_ (Figure S7).

The above results were contrasted by relatively poor agreements between ISORROPIA and measured pH, which are arising from both the differences in calculated *m*_H^+^_ in the high *m*_NaHSO_4__ range and the assumption of γ_H^+^_. The differences (ISORROPIA–experiment) of log_10_*m*_H^+^_ were in the range from −0.2 to 0.05, which is comparable to that of ACCENT results. For γ_H^+^_, since ISORROPIA calculates binary mean activity coefficients and does not provide γ_H+_,^16^ it has been widely set as unity in previous studies.^64−66^ However, γ_H^+^_ being set as unity largely deviated from experiment-based γ_H^+^_ (0.83 ≤ Δlog_10_γ_H^+^_ ≤ 0.19), resulting in pH differences up to 1.0 pH unit (range of −0.24 to 1.0 pH units) (Figures 4 and S6c).

### Global Impact of HSO_4_^–^–SO_4_^2–^ Equilibrium

**Figure 5:**
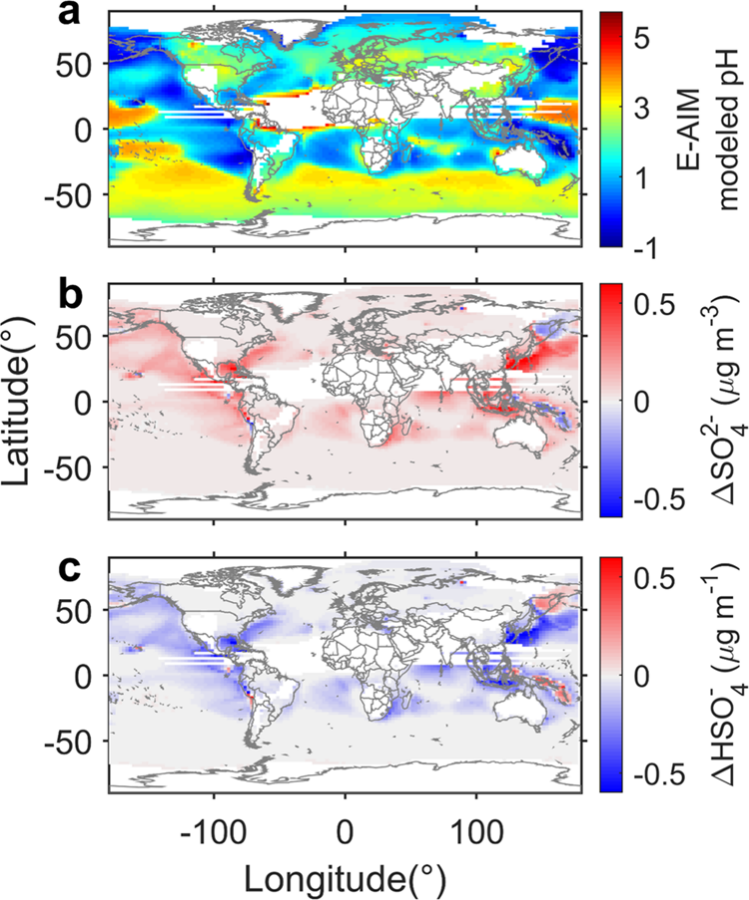
Impact of HSO_4_^–^–SO_4_^2–^ equilibrium. Global distributions of (a) PM_2.5_ pH determined from E-AIM, (b) difference in ISORROPIA–E-AIM PM_2.5_ SO_4_^2–^ concentrations (ΔSO_4_^2–^, with unit of μg m^–3^), and (c) difference in ISORROPIA–E-AIM PM_2.5_ HSO_4_^–^ concentrations (ΔHSO_4_^–^, with a unit of μg m^–3^).

Our results show that compared with ISORROPIA, E-AIM shows a much better performance in solving HSO_4_^–^ dissociation equilibrium and agrees well with measured *m*_SO_4_^2–^_ and *m*_HSO_4_^–^_ over a wide *m*_NaHSO_4__ range (0.4–8.8 mol kg^–1^), with only small average relative deviations of 12.4% for *m*_SO_4_^2–^_ and 6.9% for *m*_HSO_4_^–^_. As the treatment of HSO_4_^–^ dissociation in thermodynamic models determines the predicted equilibrium concentrations of SO_4_^2–^ and HSO_4_^–^, we performed the global model simulations to investigate the deviations of ISORROPIA for SO_4_^2–^ and HSO_4_^–^ predictions using E-AIM model results as reference. As shown in Figure 5, substantial deviations of SO_4_^2–^ and HSO_4_^–^ concentrations appear in acidic regions with aerosol pH ranging from −1 to 1, which is reasonable as little HSO_4_^–^ can exist in aerosols with higher pH. This pH range of −1 to 1 is comparable to our measured range (pH ranging from −1.15 to 0.95), and the area with such aerosol acidity accounts for ∼20% of the surface atmosphere globally in Figure 5a, as E-AIM (version IV) cannot complete the calculation of pH and ion concentrations of global aerosols when the RH is less than 60% and/or the temperature is lower than 263.15 K. The actual contribution of high acidic regions to the global surface can reach up to ∼40% when considering the ISORROPIA results alone (Figure S8). In such acidic regions, the concentration difference between the two models for both ions can reach up to 0.5 μg m^–3^ (Figure 5b,c) and the average relative deviations of ISORROPIA from E-AIM are ∼25% (ranging from 1 to 70%) for SO_4_^2–^ concentration and ∼65% for HSO_4_^–^ concentration (ranging from 1 to 100%) (Figure S9). The large deviations of SO_4_^2–^ and HSO_4_^–^ concentrations can give rise to considerable differences in H^+^ concentrations, and consequently aerosol pH up to 1 unit (Figure S10), which may largely influence the secondary organic aerosol formation due to the different reactivity of SO_4_^2–^ and HSO_4_^–^,^67,68^ as well as aerosol hygroscopicity. Note, that ISORROPIA is geared toward chemical transport modeling and efficient calculations, so its parameterization can come at the cost of accuracy. Given that ISORROPIA is computationally efficient and has been widely incorporated in global and regional air quality models^11,15^ and the importance of accurately simulating aerosol pH and the HSO_4_^–^–SO_4_^2–^ equilibrium, we would recommend optimizing ISORROPIA by updating its reactivity coefficient lookup table related to SO_4_^2–^ and HSO_4_^–^ in the future.

## Supporting Information Available

Determination of ion concentrations from experiment-based ion activity coefficients; *m*_NaHSO_4__ determined by different methods; relative deviation of *m*_SO_4_^2–^_ and *m*_HSO_4_^–^_ calculated by different models relative to the respective measurement values; differences in pH, log_10_*m*_H^+^_ and log_10_γ_H^+^_ between model calculations and experimental results; global distributions of PM_2.5_ pH determined from ISORROPIA; relative deviations of ISORROPIA from E-AIM for global PM_2.5_ SO_4_^2–^ concentration and HSO_4_^–^ concentration; difference in global PM_2.5_ pH between ISORROPIA and E-AIM (PDF)
