# Supplementary material for: Aerosol pH and Ion Activities of HSO4– and SO42– in Supersaturated Single Droplets
Source: Environ Sci Technol. 2022 Sep 1;56(18):12863–72. doi: 10.1021/acs.est.2c01378 (PMC9494740; doi:10.1021/acs.est.2c01378)
Supplement: Supplementary file 1 — es2c01378_si_001.pdf [file es2c01378_si_001.pdf]

Supplementary Information for

Aerosol pH and ion activities of HSO_4^- and SO_4^{2-} in supersaturated single droplets

Meng Li[1], Hang Su[2]*, Guangjie Zheng[1], Uwe Kuhn[2], Najin Kim[2], Guo Li[1], Nan Ma[1,3], Ulrich Pöschl[2] and Yafang Cheng[1]*

[1]Minerva Research Group, Max Planck Institute for Chemistry, 55128 Mainz, Germany; [2]Multiphase Chemistry Department, Max Planck Institute for Chemistry, 55128 Mainz, Germany; [3]Institute for Environmental and Climate Research, Jinan University, Guangzhou, 511443, China.

*Correspondence to: Y.C. (yafang.cheng@mpic.de) and H.S. (h.su@mpic.de)

13 pages

10 Figures

Text S1. The explanation of apparent inconsistency between Figure 2 and Figure S2

In the low $m_{\mathrm{NaHSO_4}}$ range (<2.0 mol kg^{-1}), although ISORROPIA predictions looked quite close to the corresponding experimental results in Figure 2b, the relative deviations reached up to 40% for $m_{\mathrm{SO_4^{2-}}}$ and 44% for $m_{\mathrm{HSO_4^-}}$ (Figure S2). This apparent inconsistency is due to ISORROPIA's insensitivity to small relative humidity (RH) changes. For example, when the input RH was in the range of 97.51%–98.43%, the outputs corresponding to different RH inputs were exactly the same for the $NaHSO_4$ system, leading to a step-like changes of $m_{\mathrm{NaHSO_4}}$ as a function of RH (Figure S3). It means that the same $m_{\mathrm{NaHSO_4}}$ value of ISORROPIA corresponds to different measured $m_{\mathrm{NaHSO_4}}$ values in this small RH range. Large ion relative deviations of ISORROPIA thus become evident since they were calculated between model calculated ion concentrations and measured ones as a function of measured $m_{\mathrm{NaHSO_4}}$ values. These large relative deviations of ISORROPIA can be easily observed when the model estimated $m_{\mathrm{SO_4^{2-}}}$ and $m_{\mathrm{HSO_4^-}}$ were related to $m_{\mathrm{NaHSO_4}}$ of the measurements (Figure S4), such is not the case in Figure 2, where the equilibrium ion concentrations were related to the $m_{\mathrm{NaHSO_4}}$ of their respective model outputs.

Text 2. Determinations of ion concentrations from experiment-based ion activity coefficients

A step-searching method was applied to calculate ion concentrations from experiment-based ion activity coefficients. The input to the method is $m_{\mathrm{Na^+}}$, which was calculated by the sum of $m_{\mathrm{SO_4^{2-}}}$ and $m_{\mathrm{HSO_4^-}}$ from measurements based on the stoichiometric formula of $NaHSO_4$. $m_{\mathrm{H^+}}$ was set in the range from 0 to the value of $m_{\mathrm{Na^+}}$ with 10000 evenly spaced steps. For each $m_{\mathrm{H^+}}$: (i) $m_{\mathrm{SO_4^{2-}}}$, $m_{\mathrm{HSO_4^-}}$,were calculated by:

$$m_{\mathrm{H^+}} = m_{\mathrm{SO_4^{2-}}} \qquad (1)$$

$$m_{\mathrm{HSO_4^-}} = m_{\mathrm{Na^+}} - m_{\mathrm{SO_4^{2-}}} \qquad (2)$$

(ii) $\gamma_{\mathrm{H^+}}$, $\gamma_{\mathrm{SO_4^{2-}}}$, $\gamma_{\mathrm{HSO_4^-}}$ were the experiment-based values calculated by their corresponding fitting equations in Figure. 3b in the main text. (iii) $K_{a,exp}$ and ΔK were calculated by:

$$K_{a,exp} = \frac{a_{\mathrm{H^+}} \cdot a_{\mathrm{SO_4^{2-}}}}{a_{\mathrm{HSO_4^-}}} = \frac{(m_{\mathrm{H^+}} \cdot \gamma_{\mathrm{H^+}})\left(m_{\mathrm{SO_4^{2-}}} \cdot \gamma_{\mathrm{SO_4^{2-}}}\right)}{m_{\mathrm{HSO_4^-}} \cdot \gamma_{\mathrm{HSO_4^-}}} \qquad (3)$$

$$\Delta K = \left|K_{a,exp} - K_a\right| \qquad (4)$$

where K_a is the HSO_4^- dissociation constant with a value of 0.01 at 298 K[1]. When ΔK reaches its minimum value and satisfies the relationship of $\frac{\Delta K}{K_a} < 0.1\%$, the corresponding ion concentrations were selected as the output. If this minimum ΔK does not meet this criterion, increase the steps until it does. The final output is $m_{\mathrm{SO_4^{2-}}}$ and $m_{\mathrm{HSO_4^-}}$.

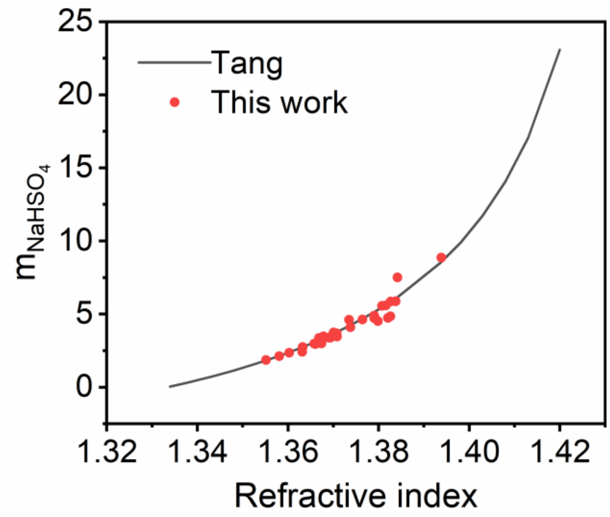

Figure S1. Total $NaHSO_4$ concentrations (m_{NaHSO_4}) determined from experimental results (red filled circle) and calculated from empirical correlations developed by Tang et al.[2] (solid line) as a function of refractive index. In AOT-Raman system, droplet refractive indexes can be directly read from the LARA software during measurements.

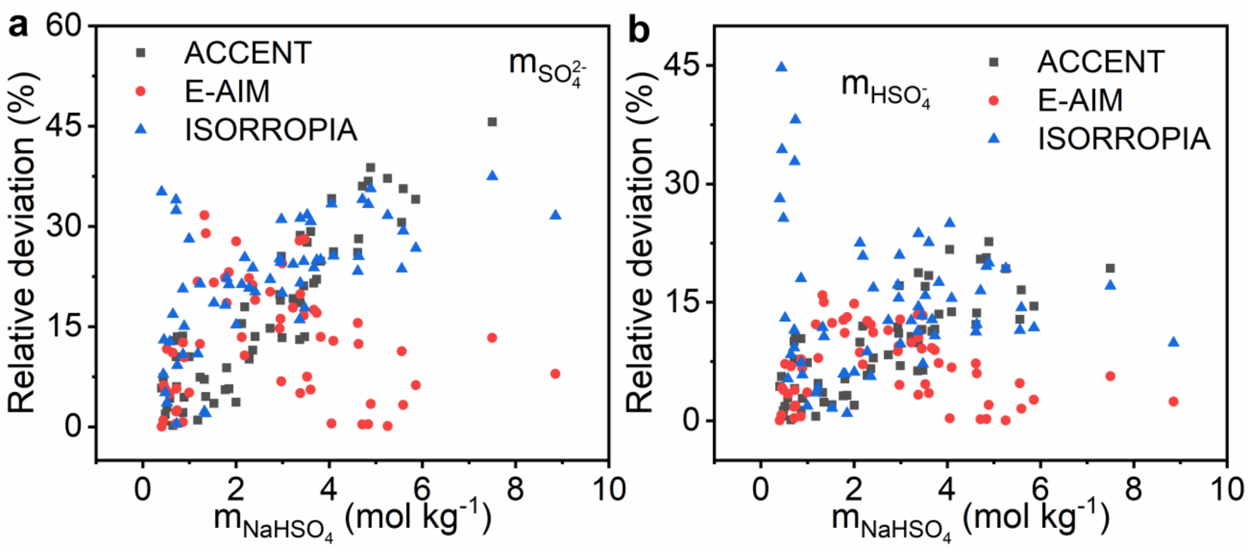

Figure S2. Relative deviations of (a) SO_4^{2-} concentrations ($m_{SO_4^{2-}}$) and (b) HSO_4^- concentrations ($m_{HSO_4^-}$) calculated with different models (black for ACCENT, red for E-AIM and blue for ISORROPIA) relative to the respective measured values as a function of m_{NaHSO_4} of measurements. Here, relative deviation=$\frac{|m_{model}-m_{measurement}|}{m_{measurement}}100\%$.

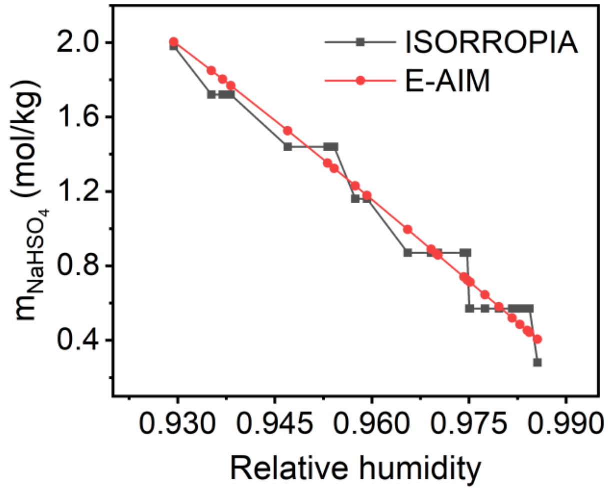

Figure S3. $m_{\mathrm{NaHSO_4}}$ estimated by ISORROPIA (black) and E-AIM (red) as a function of relative humidity.

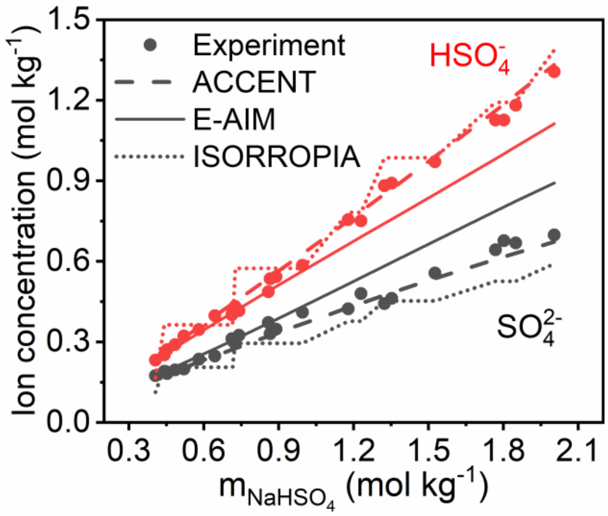

Figure S4. $m_{SO_4^{2-}}$ (black) and $m_{HSO_4^-}$ (red) determined from direct droplet measurement (solid circle), E-AIM (solid line), ACCENT (dashed line) and ISORROPIA (dotted line) as a function of measured m_{NaHSO_4}.

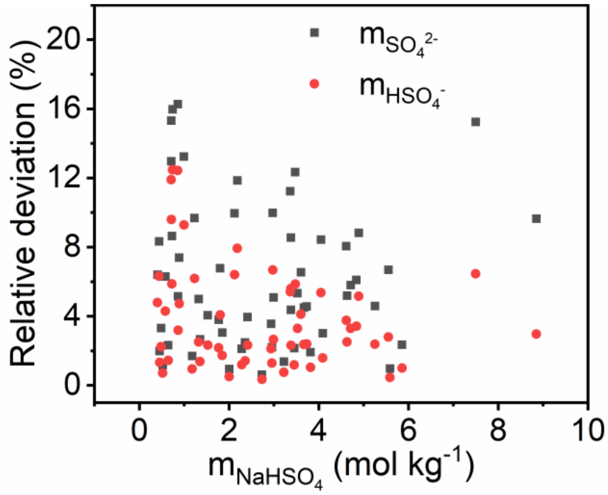

Figure S5. Relative deviations of $m_{\mathrm{SO_4^{2-}}}$ (black square) and $m_{\mathrm{HSO_4^-}}$ (red circle) calculated by the experiment-based ion activity coefficients relative to the respective measured values as a function of measured $m_{\mathrm{NaHSO_4}}$. Here, relative deviation=$\frac{|m_{calculated}-m_{measurement}|}{m_{measurement}}$100%.

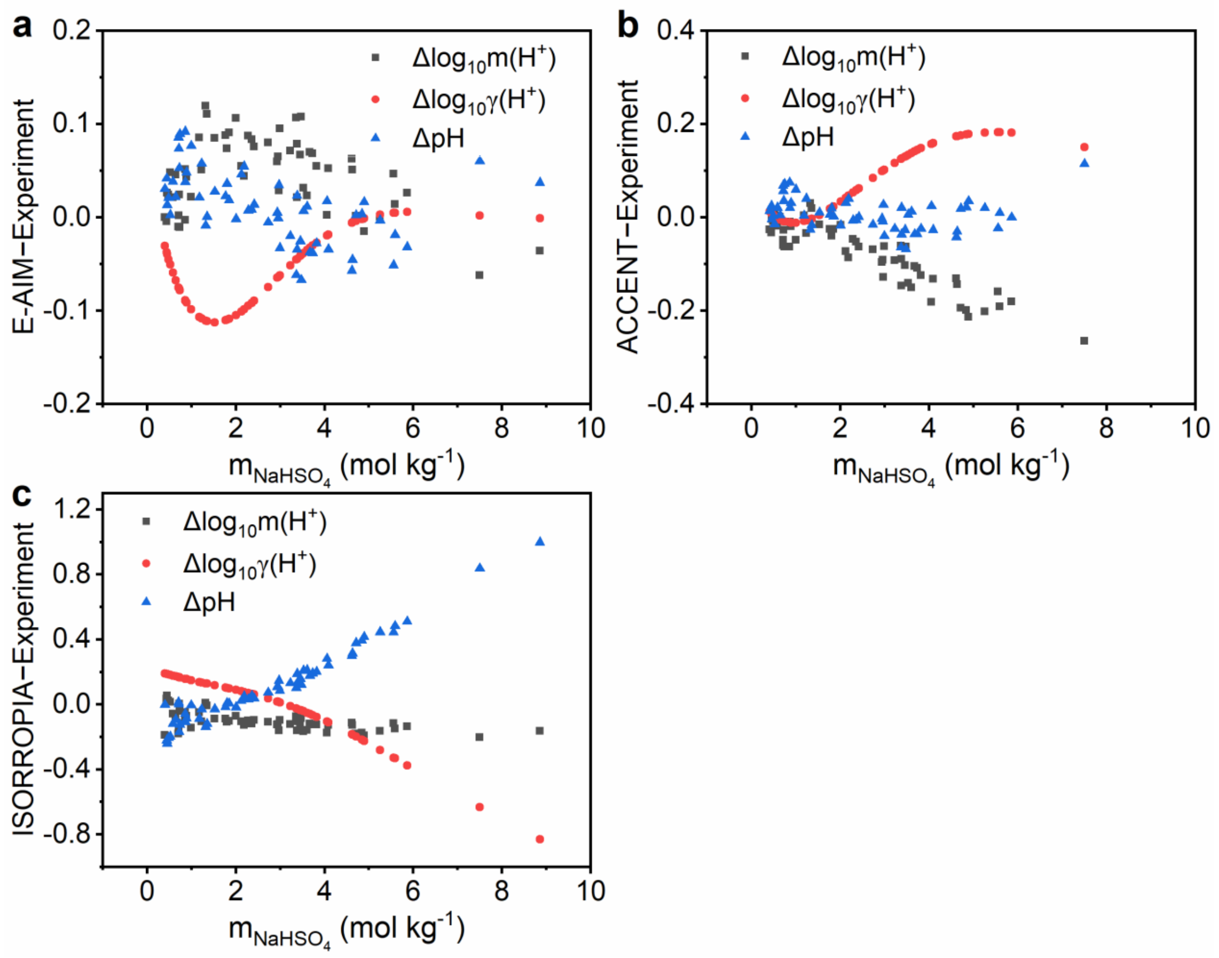

Figure S6. Differences in pH (ΔpH), $\log_{10} m_{H^+}$ ($\Delta\log_{10} m_{H^+}$) and $\log_{10}\gamma_{H^+}$ ($\Delta\log_{10}\gamma_{H^+}$) between model calculations and experiment results. (a) E-AIM − Experiment, (b) ACCENT−Experiment, (c) ISORROPIA−Experiment as a function of m_{NaHSO_4} of measurements.

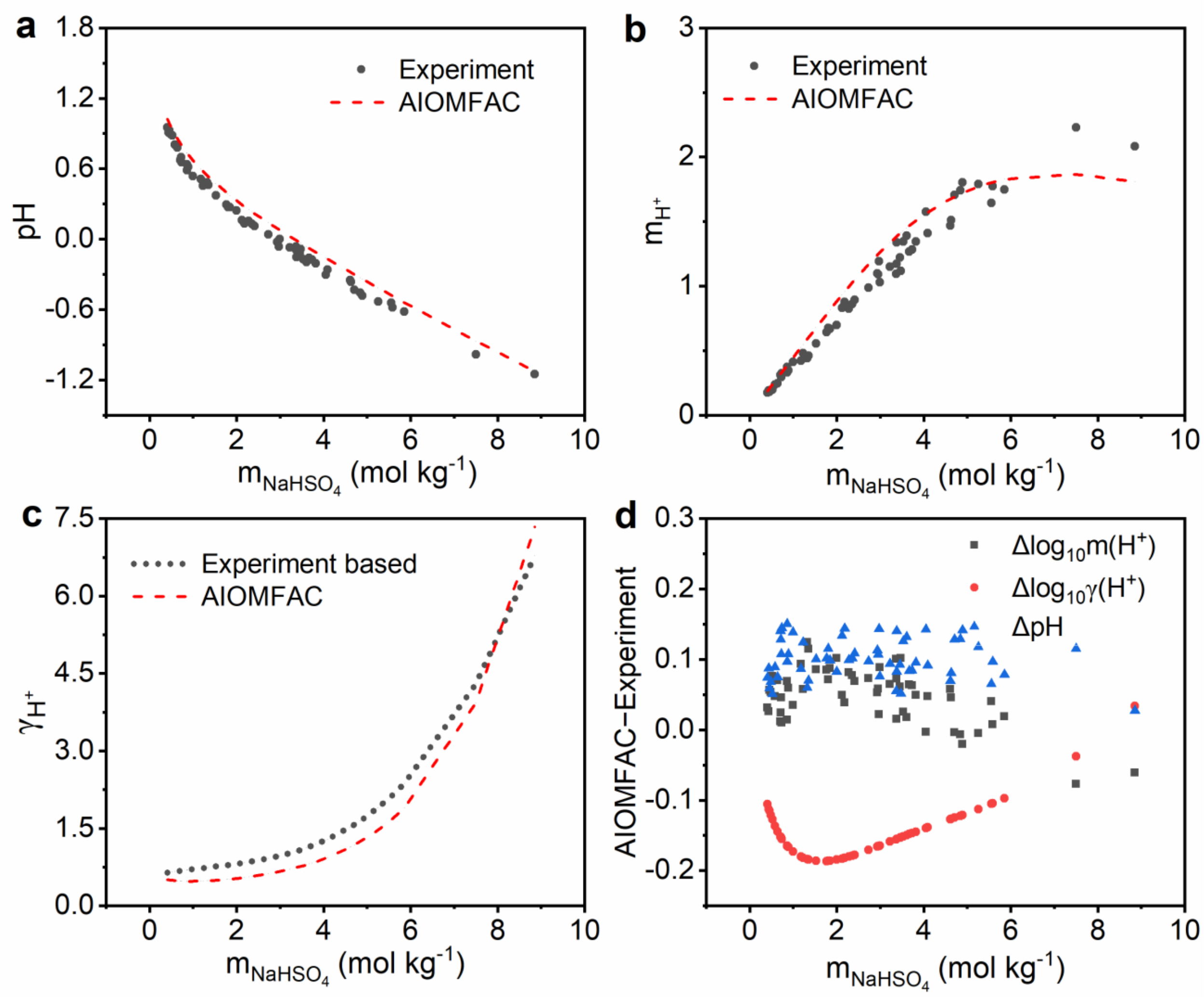

Figure S7. Comparison of observed and AIOMFAC calculated (a) pH, (b) m_{H^+} and (c) γ_{H^+}. (d) ΔpH, $\Delta\log_{10}m_{\mathrm{H}^+}$ and $\Delta\log_{10}\gamma_{\mathrm{H}^+}$ between AIOMFAC calculations and experimental results (AIOMFAC−Experiment).The inputs for AIOMFAC (https://aiomfac.lab.mcgill.ca/index.html) were the temperature (298.15K) and the mole fraction of $NaHSO_4$ (calculated from the molality of $NaHSO_4$).

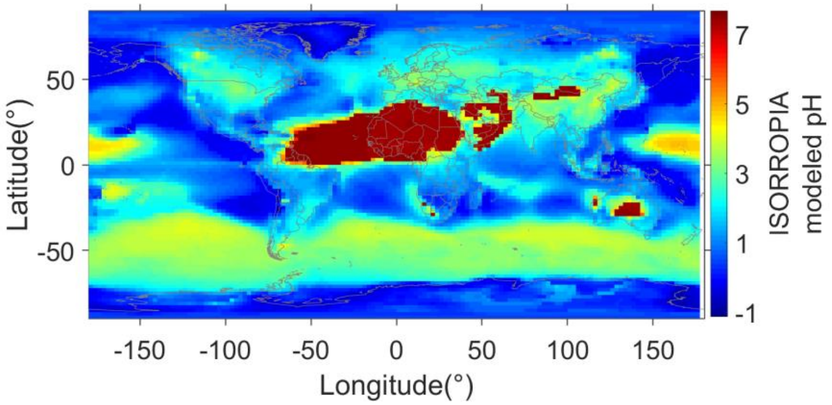

Figure S8. Global distributions of $PM_{2.5}$ pH determined from ISORROPIA.

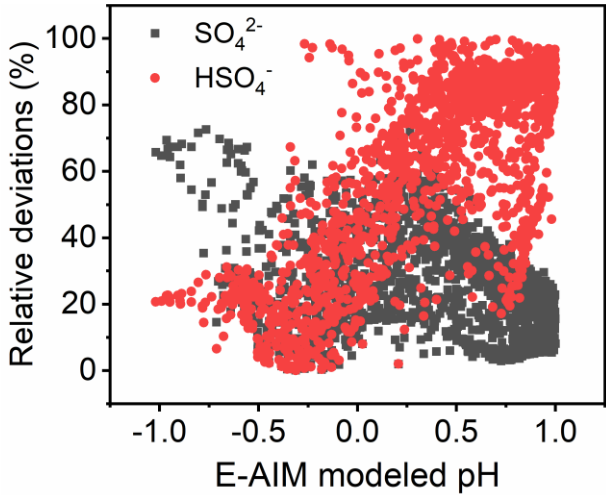

Figure S9. Relative deviations of global $PM_{2.5}$ SO_4^{2-} concentrations (black) and HSO_4^- concentrations (red) as a function of E-AIM modeled global aerosol pH in highly acidic environments (pH ranging from -1 to 1). Here, relative deviation=$\frac{|\text{ISORROPIA}-\text{E}-\text{AIM}|}{\text{E}-\text{AIM}}100\%$.

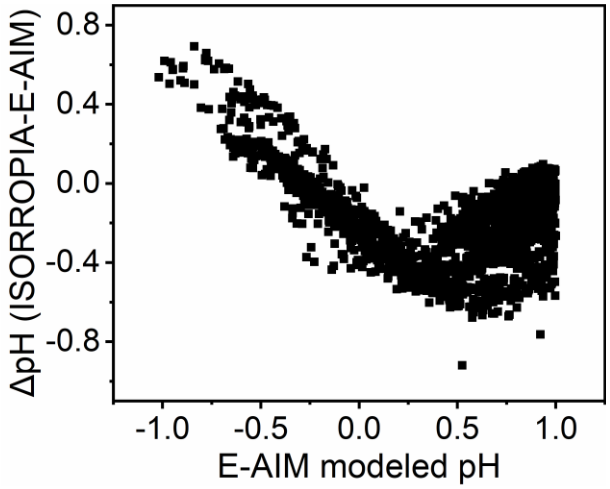

Figure S10. Global ΔpH between ISORROPIA and E-AIM as a function of E-AIM modeled global $PM_{2.5}$ pH in highly acidic environments (pH ranging from -1 to 1).

Reference

(1) Rindelaub, J. D.; Craig, R. L.; Nandy, L.; Bondy, A. L.; Dutcher, C. S.; Shepson, P. B.; Ault, A. P. Direct measurement of pH in individual particles via Raman microspectroscopy and variation in acidity with relative humidity. *J. Phys. Chem. A* **2016**, *120* (6), 911-917.

(2) Tang, I. N.; Munkelwitz, H. R. Water activities, densities, and refractive indices of aqueous sulfates and sodium nitrate droplets of atmospheric importance. *J. Geophys. Res. Atmos.* **1994**, *99* (D9), 18801-18808.
